# Diversity and genetics of nitrogen-induced susceptibility to the blast fungus in rice and wheat

**DOI:** 10.1186/1939-8433-6-32

**Published:** 2013-11-20

**Authors:** Elsa Ballini, ThuyThu Thi Nguyen, Jean-Benoit Morel

**Affiliations:** SupAgro, UMR BGPI INRA/CIRAD/SupAgro, Campus International de Baillarguet, TA A 54/K, Montpellier, 34398 France; INRA, UMR BGPI INRA/CIRAD/SupAgro, Campus International de Baillarguet, TA A 54/K, Montpellier, 34398 France

**Keywords:** Rice, Wheat, *Magnaporthe oryzae*, Nitrogen, NUE

## Abstract

**Background:**

Nitrogen often increases disease susceptibility, a phenomenon that can be observed under controlled conditions and called NIS, for Nitrogen-Induced Susceptibility. NIS has long been reported in the case of rice blast disease caused by the fungus *Magnaporthe oryzae.* We used an experimental system that does not strongly affect plant development to address the question of NIS polymorphism across rice diversity and further explored this phenomenon in wheat. We tested the two major types of resistance, namely quantitative/partial resistance and resistance driven by known resistance genes. Indeed there are conflicting reports on the effects of NIS on the first one and none on the last one. Finally, the genetics of NIS is not well documented and only few loci have been identified that may control this phenomenon.

**Results:**

Our data indicate that NIS is a general phenomenon affecting resistance to blast fungus in these two cereals. We show that the capacity of rice to display NIS is highly polymorphic and does not correlate with difference related to indica/japonica sub-groups. We also tested the robustness of three different major resistance genes under high nitrogen. Nitrogen partially breaks down resistance triggered by the *Pi1* gene. Cytological examination indicates that penetration rate is not affected by high nitrogen whereas growth of the fungus is increased inside the plant. Using the CSSL mapping population between Nipponbare and Kasalath, we identified a Kasalath locus on chromosome 1, called *NIS1*, which dominantly increases susceptibility under high nitrogen. We discuss the possible relationships between Nitrogen Use Efficiency (NUE), disease resistance regulation and NIS.

**Conclusions:**

This work provides evidences that robust forms of partial resistance exist across diversity and can be easily identified with our protocol. This work also suggests that under certain environmental circumstances, complete resistance may breakdown, irrelevantly of the capacity of the fungus to mutate. These aspects should be considered while breeding for robust forms of resistance to blast disease.

**Electronic supplementary material:**

The online version of this article (doi:10.1186/1939-8433-6-32) contains supplementary material, which is available to authorized users.

## Background

Plant disease resistance is often affected by high nitrogen supply. More generally, the effects of high nitrogen on plant disease are depending on the plant-host combination (Dordas 2008). For instance, it is commonly observed that high nitrogen enhances susceptibility to biotrophic and hemibiotrophic pathogens while it reduces it in the case of necrotrophic pathogens. However in some cases, the growth of pathogens that are considered as hemibiotrophic is reduced under high nitrogen. This is the case of the interaction between wheat and *Fusarium graminearum* (Yang et al. 2010).

In the case of rice blast disease caused by the *Magnaporthe oryzae* fungus, early reports mention this phenomenon as « Koe-imochi » (Bonman 1992; Otani 1959; Ou 1972; Tanaka 1961). The molecular and cytological mechanisms of this phenomenon that we call Nitrogen-Induced Susceptibility (NIS) are far from being understood. Two major hypotheses can be proposed to explain this phenomenon: the existence of a trophic relationship impacting on fungal pathogenicity and/or molecular interactions between nitrogen metabolism and disease resistance pathways. Here we show that NIS also exists in the case of the wheat blast disease and we explore the genetic diversity and determinism of NIS in rice.

To defend themselves against pathogen attacks, plants have developed defense systems that are articulated in three major steps: recognition by different types of receptors, signal transduction and finally induction of the defense arsenal including for example the Pathogenesis-Related (*PR*) genes (Jones and Dangl 2006). When a particular motif from the pathogen is recognized by such a plant receptor, the plant efficiently triggers inducible systems that lead to resistance. In that situation, the interaction is called incompatible, the resistance is complete, the receptor is defined as a resistance (*R*) gene and the pathogen is avirulent. This type of resistance is associated with the development of the so-called Hypersensitive-response (HR) that is often manifested by visible necrotic lesions (later called resistant lesions). Breeders have long been using this type of *R* genes in their breeding schemes; indeed *R* genes are usually dominant and they confer strong levels of resistance. However, the drawback of such genes is that they are often not durable because pathogens can evolve to by-pass recognition by the *R* genes (Correa-Vitoria and Zeigler 1994; Boyd et al. 2013). To our knowledge, the efficiency of *R* genes under high nitrogen has not been studied.

Breeders have also been using another form of resistance called partial or quantitative resistance. A partial resistance genotype allows limited but significantly reduced pathogen reproduction when compared with a susceptible genotype (Parlevliet and van Ommeren 1975). Partial resistance, while conferring lower levels of resistance, is usually considered as broad-spectrum, although this assumption is not always based on the observation (Ballini et al. 2008). Lesion density and diseased surface are often considered as the major traits defining partial resistance (Talukder et al. 2005; Roumen 1994). Whereas lesion density usually reflects the success of penetration of the pathogen, the diseased surface is mostly conditioned by the capacity of the pathogen to progress inside the plant tissues. In the field, partial resistance to rice blast was shown to be inefficient under high nitrogen (Prabhu et al. 1996) whereas Talukder et al. (2005) observed under controlled conditions that partial resistance is not strongly affected. Thus there is yet no consensus on the extent of NIS in the case of partial resistance to blast disease. Furthermore, there has been no examination of NIS across a representative rice diversity panel.

There is only one report on the genetic analysis of the effect of nitrogen on blast resistance (Talukder et al. 2005). In this work the authors analyzed a recombinant population between the Azucena and Bala genotypes. They found three loci conferring partial resistance only under low nitrogen input. In contrast, four partial resistance loci were robust under high nitrogen and one was only observed under high nitrogen. The genetic maps produced were not detailed enough to allow speculations on the genes potentially responsible for the phenotypes observed. Our knowledge on the genetic control of NIS is thus very limited and identifying loci responsible for this effect should shed some light on this phenomenon.

In this work, we addressed these questions related to the diversity and genetic control of NIS in rice. Using our protocol of nitrogen-induced susceptibility, we tested whether the observation of this phenomenon could also be extended to another cereal, wheat, that is attacked by the blast fungus *M. oryzae*. Using this disease as a model, NIS was measured in a panel of representative rice cultivars and in wheat. We also evaluated the robustness of known resistance genes under high nitrogen and analyzed how disease resistance was triggered at the molecular level in these conditions. Furthermore, we initiated a genetic mapping of NIS in rice to identify loci that are important for the control of this phenomenon.

## Results

### Nitrogen-induced susceptibility across rice diversity

**Figure 1:**
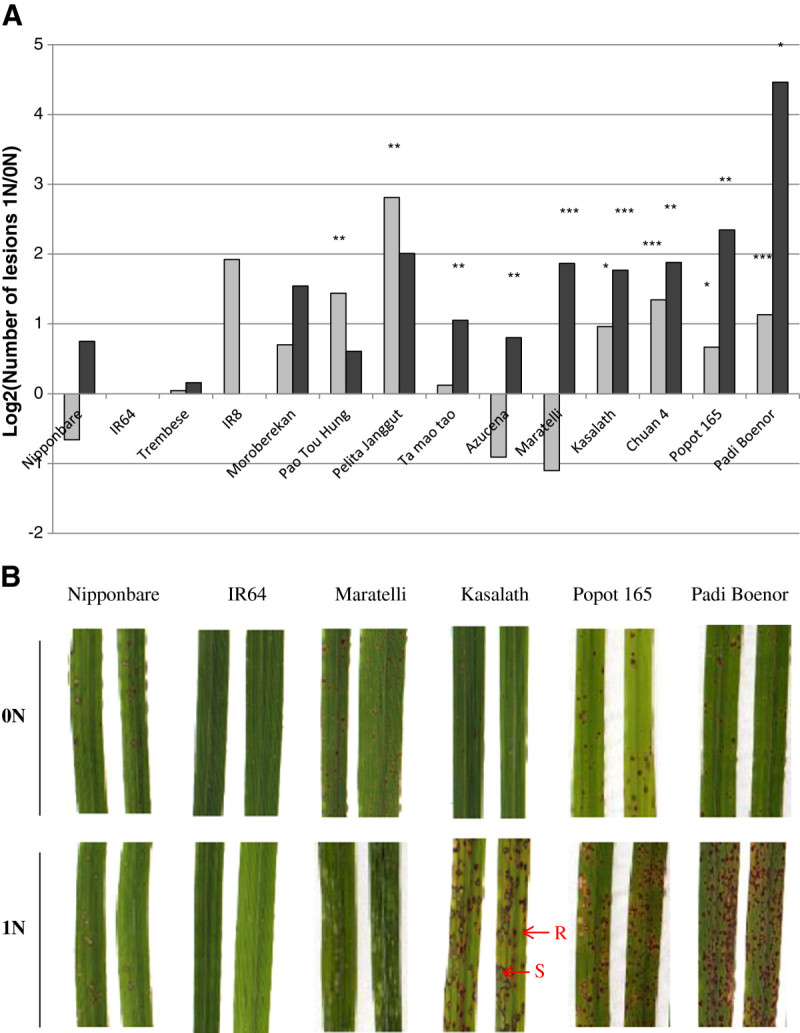
**Diversity of nitrogen-induced susceptibility in rice.** Nitrogen (1 N) or no nitrogen (0 N) was added to rice plants one day before inoculation with the GUY11 isolate of *M. oryzae* (see Methods). **A.** Different types (shown in B) of lesions typical of partial resistance were counted 7 days post-inoculation (dpi): lesions typical of resistance (light grey bars) and lesions typical of susceptibility (dark grey bars). The mean of each category of lesion was compared between the 0 N and 1 N condition and statistical differences are shown by *** (p value < 0.001; Wilcoxon test). **B.** Examples of nitrogen-induced susceptibility at 7 dpi. Lesions typical of resistance (R) and lesions typical of susceptibility (S) are shown.

Nitrogen enhances susceptibility in a simplified system involving rice and *M. oryzae*. In his pioneer experiments, Otani (1959) tested the effect of source and dose of nitrogen supply at different times before inoculation with rice blast. Based on his experiment we have elaborated a protocol to test for the effect of a high level of nitrate or ammonia fertilization added one day before inoculation with *M.oryzae*. We next tested intra-specific diversity of this phenomenon in rice. We thus evaluated the level of polymorphism of NIS across rice diversity. This evaluation is critical for both the identification of robust forms of disease resistance as well as for the identification of loci important for robustness. We used a set of 14 lines representative of rice diversity and well-characterized for their quantitative levels of resistance towards *M. oryzae* (Vergne et al. 2010). This represents 7 indica and 7 japonica types (Additional file 1: Table S1) that were grown under similar nitrogen regimes until the fourth leave had emerged. One day before inoculation, high or low nitrogen inputs were added (see Methods). Partial resistance is manifested by two major types of lesions (Roumen 1994) (see arrows in Figure 1B): grayish lesions, in the center of which the fungus sporulates (susceptible lesion) and small brown lesions indicative of successful resistance reaction (resistant lesion). Symptoms were noted and both types of lesions were counted (Figure 1) seven days after inoculation with the GUY11 isolate. A first group of seven cultivars (Padi Boenor, Kasalath, Popot 165, Chuan 4, Azucena, Maratelli and Ta Mao Tao) showed increased susceptibility to *M. oryzae* upon nitrogen supply (Figure 1A) with the least robust genotype being Padi Boenor. Two sub-classes of phenotypes could be further distinguished in this group: the Azucena, Maratelli and Tao Mao Tao cultivars only showed a significant increase of susceptible lesions while the other cultivars showed an increase of susceptible lesions but also resistant ones (see below). Because both indica and japonica types are found in this group of cultivars displaying NIS, this genetic difference does not seem to be responsible for the observed differences in robustness to nitrogen-induced susceptibility. The breakdown of partial resistance was also observed with three other *M. oryzae* isolates (Additional file 1: Table S1), suggesting that this nitrogen-induced susceptibility is not isolate specific and affects about half of our diversity panel.

A second group of seven cultivars (IR64, IR8, Moroberekan, Nipponbare, Trembese and Pao Tu Hung and Pelita Janggut) showed robust resistance to *M. oryzae*. Two cultivars (Pao Tou Hung and Pelita Janggut) showed a significant increase in resistant lesions but overall the plants remained resistant. In most cases, the cultivars tested showed high starting levels of resistance, which is hypothesized to result from a combination of major resistance genes and high partial resistance level (Wang et al. 1994; Roumen 1994). Thus it is possible to find genotypes that display robust resistance levels even under high nitrogen supply. This confirms similar results observed previously in the field (Long et al. 2000).

### Nitrogen supply and major resistance genes

**Figure 2:**
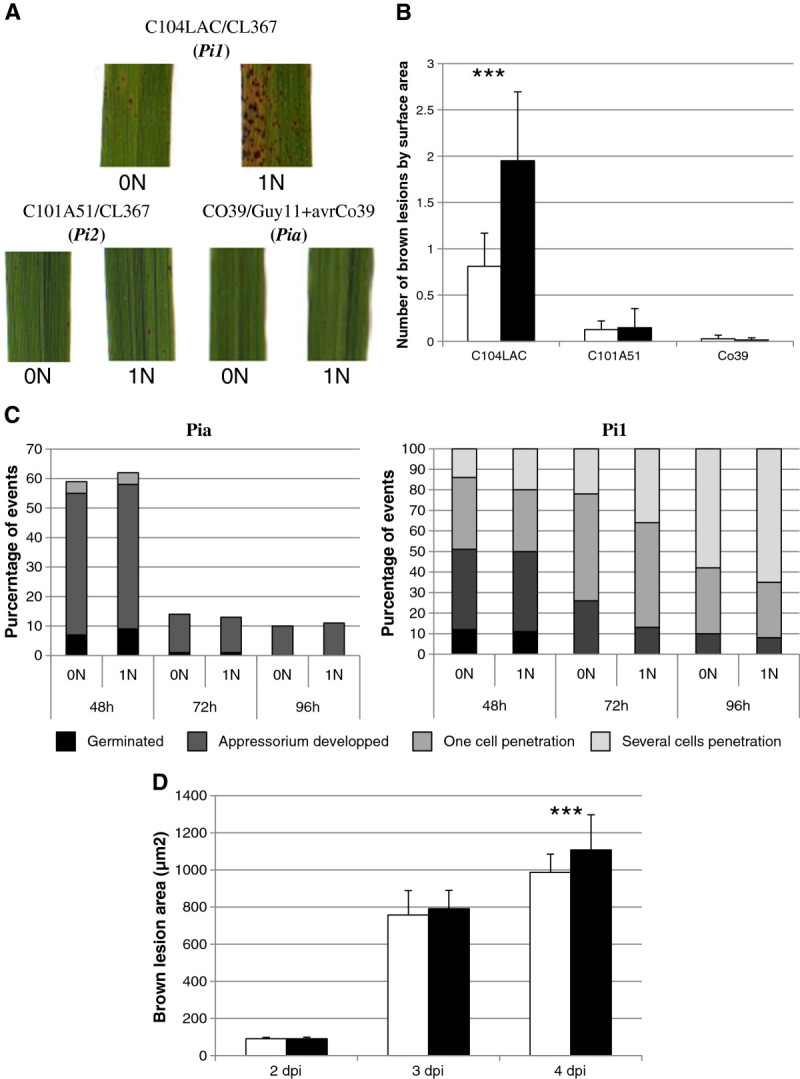
**Robustness of resistance genes under high nitrogen treatment.** Nitrogen (1 N) or no nitrogen (0 N) was added to rice plants one day before inoculation (see Methods). **A.** Different resistance genes (*Pi1*, *Pi2* and *Co39*/*Pia*) were tested using different combinations of rice cultivars and *M. oryzae* isolates. **B.** The number of lesions typical of resistance was counted. There were no susceptible lesions under these conditions (white bars: 0 N; black bars: 1 N). The mean of the number of resistant lesions were compared between the 0 N and 1 N conditions and statistical differences are shown by *** (p value < 0.001; Wilcoxon test). **C.** At the indicated time points after inoculation, the development stage of the fungus was observed and four categories of growth stages were counted 100 interaction sites/condition). This experiment was repeated 3 times and gave similar results. **D.** The average size of individual fungal colonies was measured inside plant tissues. The mean and sd are shown 50 colonies counted for each condition; white bars: 0 N and black bars: 1 N). Statistical differences are shown by *** (p value < 0.001; Wilcoxon test).

The increased number of the resistant lesions in some rice lines (Figure 1) upon high nitrogen supply prompted us to evaluate whether the triggering of resistance by known major resistance (*R*) genes could be affected. For that purpose we tested three *R* genes: *Co39/Pia* (Chauhan et al. 2002; Okuyama et al. 2011), *Pi1* (Hua et al. 2012) and *Pi2* (Zhou et al. 2006). The *Co39* and *Pia* genes are allelic, code for a duplex of NBS-LRR proteins and are revealed in the interaction between cultivar CO39 and the GUY11 transgenic isolate expressing the AvrCo39 avirulence gene (Cesari et al. 2013). The *Pi2* (revealed in the interaction between C101A51 line and CL367 isolate; (Telebanco-Yanoria et al. 2011)) gene codes for an NBS-LRR paralogue of the *Pi9* loci (Zhou et al. 2006). The *Pi1* gene (involved in the interaction between the cultivar C104Lac and the isolate CL367 (Telebanco-Yanoria et al. 2011) codes for an NBS-LRR (Hua et al. 2012). The *Co39*/*Pia* and *Pi2* genes triggered resistant lesions to the same level irrelevantly of the nitrogen treatment (Figures 2A and B). Thus there are major resistance genes that do not seem to be affected by nitrogen supply.

In contrast, the number of resistant lesions triggered by the *Pi1* gene significantly increased under high nitrogen regime as shown in the C104Lac line (Figures 2A and B), although the plants did not die or develop disease symptoms. We hypothesized that the weakening of resistance triggered by high nitrogen supply increased the number of plant cells exposed to the fungus and that consequently the setting of defense reactions in an increased number of cells could lead to an increase of visible necrosis. Whether this temporary weakening of *R* gene-triggered resistance occurs before or after penetration was tested. Using confocal microscopy, we measured at the cellular level the growth of *M. oryzae* in the contrasted cases of *Pi1* and *Co39*/*Pia* (see Methods). As to differentiate between 0 N and 1 N conditions, we calculated the percentage of each stage of growth classically used to monitor *M. oryzae*’s growth: spore germination, appressorium formation, one-cell penetration and penetration in more than one cell (Delteil et al. 2012). In the case of the *Pi1* gene, we observed no difference in penetration at 48 hours post inoculation (hpi) between high and low nitrogen regime (Figure 2C). Thus a modification of the penetration rate is unlikely responsible for the observed increase in resistant lesions. After penetration, there was a transient and slight increase in growth of the fungus at 72 hpi (Figure 2C). We further measured the growth of individual colonies (Figure 2D). At 96 hpi the average size of *M. oryzae* colonies was significantly higher under high nitrogen than under low nitrogen supply. Altogether, these data suggest that at least temporarily, the growth of the fungus inside the plant is not as halted by *Pi1* under high nitrogen than it is under low nitrogen input.

**Figure 3:**
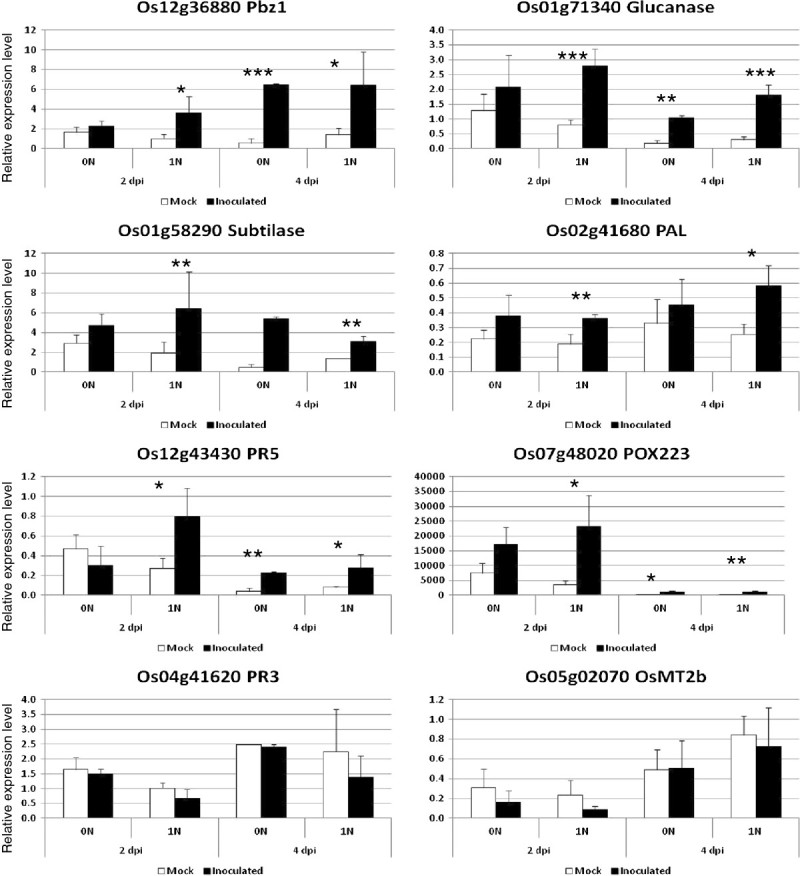
**Defense gene expression triggered by the** ***Pi1*** **resistance gene under nitrogen treatment.** Rice plants of the C104Lac genotype (containing the *Pi1* resistance gene recognizing the avirulent isolate of *M. oryzae* CL367) were grown under the same conditions except one day before inoculation where they were either treated with no additional nitrogen (0 N) or additional nitrogen (1 N). Uninfected plants (white bars: Mock) were used as controls to compare to infected plants (black bars). The expression of defense-related marker genes (normalized with the actin gene; arbitrary units are given) was measured at the indicated time points after infection (2 and 4 days post inoculation-dpi). The mean and sd of three independent replicates are shown. The statistical differences between mock and inoculated plants are shown (Wilcoxon tests; *: p < 0.05; **p < 0.01; ***: p < 0.001).

We next evaluated whether enhanced defense response could be associated with the increase of resistant lesion upon nitrogen stress. The transcription of 8 classical defense genes (Delteil et al. 2012) was measured by quantitative RT-PCR to see how high nitrogen affects the triggering of defense by the *Pi1* resistance gene. Except for the mRNA of *OsMT2b* and *PR3* genes that were not differentially expressed, and for the subtilase that is induced in both treatments, the five remaining genes were all induced earlier and/or stronger under high nitrogen (Figure 3). This suggests that the transcription of some known defense systems is strongly induced by the *Pi1* gene under high nitrogen supply.

### Genetic analysis of nitrogen-induced susceptibility

As a first step towards identifying genetic components responsible for nitrogen-induced susceptibility, we initiated a genetic analysis using a Chromosomal Segment Substitution Lines (CSSL) from a cross between Nipponbare and Kasalath. These two parents were chosen as they are polymorphic for their response to *M. oryzae* under nitrogen pressure (Figure 1B and Additional file 1: Figure S1). These CSSL lines have been previously used to decipher complex traits (Wan et al. 2006). Because the recurrent parent of this population is Nipponbare, the expectation is that the majority of the lines should display robust partial resistance under high nitrogen. Conversely, the lines where a Nipponbare chromosomal segment has been replaced by an hypothetically non-functional allele from Kasalath are expected to display the Kasalath parental phenotype (breakdown of partial resistance under high nitrogen). We used the GUY11 isolate to screen CSSL lines under high and low nitrogen supply. Out of the 54 lines tested, 38 showed robust resistance under high nitrogen and 16 showed to some extent a breakdown of partial resistance under high nitrogen input. Of these 16, three lines (CSSL4, CSSL5 and CSSL19) reproducibly showed elevated NIS for the GUY11 isolate and also for five other *M. oryzae* isolates tested (Additional file 1: Figure S1 and S2) representative of a large diversity of the fungus (Tharreau D., pers. Comm.). We focused on the further analysis of these three lines.

**Figure 4:**
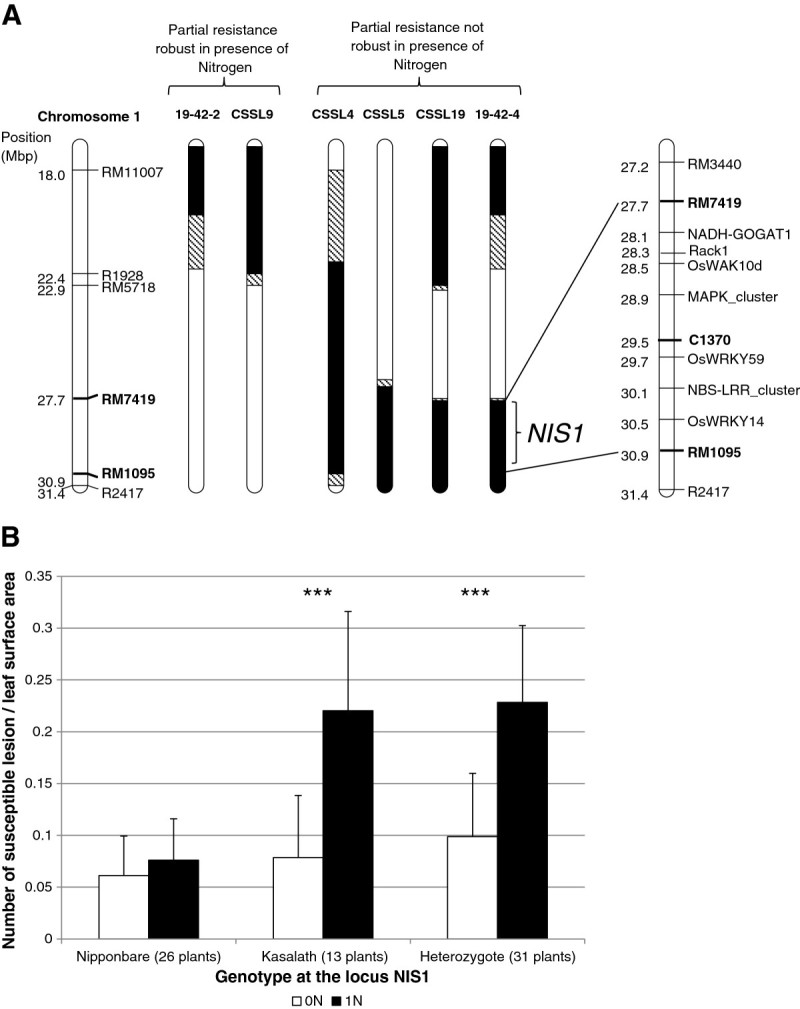
**Mapping of a locus required for resistance under high nitrogen.** The Nipponbare X Kasalath CSSL mapping population was scored for NIS to the GUY11 isolate (see Methods). **A.** The CSSL5, CSSL19 lines and two recombinant lines (19-42-2 and 19-42-4) identify chromosome 1 as carrying a Kasalath locus conferring susceptibility under high nitrogen treatment. The portions of the chromosome in black, white and dashed respectively correspond to Kasalath, Nipponbare and heterozygous as established by microsatellites markers (RM markers indicated on the left of the figure). Possible regulators of disease resistance and nitrogen metabolism are indicated for the *NIS1* locus on the right of the figure. **B.** Plants from the 19–42 family segregating at the *NIS1* locus were grown under low (0 N) or high nitrogen (1 N) and genotyped with several markers flanking the *NIS1* locus. The genotype at *NIS1* is indicated. Quantitative phenotype of these group of plants with similar genotype (the number of individual plants is indicated) was established after inoculation with the GUY11 isolate. Statistical differences are shown by *** (p value < 0.001; Wilcoxon test).

Comparing the published genetic maps of the CSSLs 4, 5 and 19 suggested that one region of chromosome 1 was common to these lines. However these lines contain several insertions of Kasalath and the phenotype could be explained by different loci in each line. Using CSSL lines that contain overlapping chromosomal substitutions but that were *not* more susceptible under high nitrogen supply, we could further eliminate segments on all the other chromosomes (Additional file 1: Table S2; data not shown). At this level of analysis, the only segment remaining as associated with the observed increase of blast susceptibility was on chromosome 1 (between RM5718 and RM1095; 8 Mb, Figure 4A).

The CSSL19 line was backcrossed to the Nipponbare parent and F3 or F4 lines were genotyped using microsatellite markers and evaluated for nitrogen-induced susceptibility to GUY11. We next performed further genetic analyses to confirm the existence of this locus and establish the heritability of the phenotype. As shown in Figure 4B, nitrogen-induced susceptibility was as high in plants heterozygous for the chromosome 1 segment than in plants homozygous for the Kasalath allele. We concluded that the nitrogen-induced susceptibility is brought in a dominant manner by the Kasalath allele.

The analysis of several F3 lines derived from a back-cross of the CSSL4 or CSSL19 lines allowed us to confirm that chromosome 1 was sufficient to confer nitrogen-induced susceptibility (Families 4–40; Additional file 1: Table S2). One F3 line (Family 19–42 and siblings; Additional file 1: Table S3) also allowed us to narrow down the interval containing the Kasalath locus responsible for this phenotype. This locus was found in a 3.2 Mb interval between RM7419 and RM1095 markers (Figure 4A) and was called *NIS1* (for nitrogen-induced susceptibility 1).

### Nitrogen-induced susceptibility and wheat blast disease

**Figure 5:**
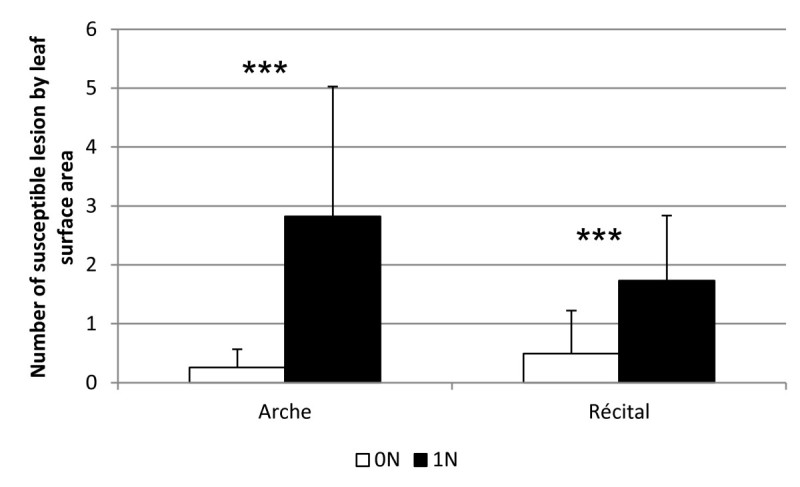
**Nitrogen-induced susceptibility to wheat blast.** Nitrogen (black bars; 1 N) or no nitrogen (white bars; 0 N) was added to wheat plants one day before inoculation with the BR32 isolate of *M. oryzae* (see Methods). Lesions were counted 7 days post-inoculation. ***: p value < 0.001 (Wilcoxon test).

Wheat blast disease, caused by isolates of *Magnaporthe oryzae* specialized on wheat has become a major threat in Southern America (Debona et al. 2012; Kohli et al. 2011). The effect of high nitrogen on the impact of wheat blast has not been evaluated thus far. We further tested whether this phenomenon could be extended to wheat blast disease. Two wheat cultivars, Arche and Récital, were tested with the *M. oryzae* BR32 isolate which is aggressive on wheat (Figure 5). The two lines tested were more susceptible to the wheat blast isolate BR32 under high nitrogen supply. Similar results were observed with another isolate (data not shown). The nitrogen-induced susceptibility to wheat blast was higher in the Arche cultivar than in the Récital cultivar. Thus NIS can also be observed in the case of wheat blast.

## Discussion

### NIS is polymorphic across rice diversity

We have shown that high nitrogen input before inoculation with *M. oryzae* increases susceptibility in the rice Kasalath genotype. This phenomenon was called NIS, for nitrogen-induced susceptibility. In this study, we asked whether NIS is affecting different forms of resistance in rice and in wheat. Our data indicate that NIS is a general phenomenon affecting resistance to blast fungus in rice (Figure 1) and wheat (Figure 5). Whether NIS in wheat reflects an agronomical situation remains to be established but our results suggest that high nitrogen input could be an additional element that favors the emergence of this disease on wheat. Moreover, it remains to be established whether our protocol for inducing NIS affects other pathogens, in particular necrotrophic ones like *Rhizoctonia solani*.

A first report of a differential cultivar response to N fertilizer in regard to rice blast was made based on field experiment (Long et al. 2000) with higher increase in disease incidence on very susceptible cultivars than in moderately susceptible cultivars. It was also reported that the Kaybonnet cultivar was a higly resistant cultivar in the presence of N fertilizer. In their study, Talukder et al. (2005) observed that the efficacy of partial resistance was not greatly affected by high nitrogen. This was based on the observation that out of the 12 disease-related QTL detected in a mapping population between Azucena and Bala, six were detected under low and high nitrogen regimes. It is noteworthy that five of these six QTLs originated from the Bala parent, indicating that Azucena and Bala do not have the same potential for displaying robust partial resistance under high nitrogen. This suggested that robustness to NIS could be polymorphic across rice diversity, an hypothesis supported by other reports (Prabhu et al. 1996). However, in most of these analyses, important growth differences provoked by different nitrogen regimes make difficult the interpretation of the phenotypes. Indeed nitrogen impacts on plant growth which in turn modifies epidemiological parameters. Here we used an experimental system that does not strongly affect plant development (data not shown) to address the question of NIS polymorphism across rice diversity. In our analysis of rice diversity (Figure 1), we show that out of the 14 rice genotypes tested, seven show robust forms of partial resistance. The quantitative levels of NIS, as measured by the increase of susceptible lesion numbers in high nitrogen condition compared to low nitrogen condition, varies from low (e.g. Azucena) to high (e.g. Padi Boenor). Thus the capacity to display NIS is polymorphic in rice. This polymorphism does not seem to relate to the indica/japonica sub-groups. We conclude that robust forms of partial resistance exist across diversity. This is supporting the observation that six of the QTL conferring partial resistance are not affected under high nitrogen (Talukder et al. 2005). Scrutinizing diversity will be important to identify robust forms of partial resistance to blast.

### NIS can impact major resistance genes

Besides partial resistance, breeders mostly use complete resistance driven by *R* genes. There has been no examination so far of the effect of nitrogen on the efficiency of these forms of resistance. Here we tested the robustness of three different *R* genes under high nitrogen. We observed that two of the *R* genes tested, *Co39/Pia* and *Pi2*, are not macroscopically affected under high nitrogen (Figure 2). In contrast, the functioning of the *Pi1* gene was visibly affected as plants showed more necrotic lesions typical of the hypersensitive response under high nitrogen. Cytological examination indicates that penetration rate is not affected by high nitrogen. Thus we can exclude the hypothesis that the penetration rate of the fungus is enhanced under high nitrogen as proposed by Talukder et al. (2005). In contrast, we observed that the fungus grew faster inside the plant under high nitrogen (Figures 2C and D). We propose that the increase of visible necrosis under these conditions is due to a mechanical side-effect of this increased growth. This may be merely due to an increase in the number of cells invaded by the fungus that in turn triggered an enhanced triggering of defense (Figure 3), including cell death, under high nitrogen than under low nitrogen. Very recently, a partial breakdown of resistance to *Pseudomonas syringae* has been observed when feeding Tobacco plants with NH4+ (Gupta et al. 2013). In that case, this breakdown of resistance was associated with a reduction of nitric oxide production, a known HR regulator, and a slight reduction of the expression of one of the eight defense-related genes tested. This case contrasts to our finding and suggests that resistance weakening in the interaction between rice and *M. oryzae* does not follow the same trend. In that respect, the example of the *Pi1* gene provides to our knowledge the first case of partial weakening of complete resistance under high nitrogen in rice. This suggests that under certain environmental circumstances, complete resistance may be weakened, irrelevantly of the capacity of the fungus to mutate.

It is noteworthy that the C104Lac line used for testing *Pi1* and the C101A51 line used to test *Pi2* are derivatives of the Co39 background used to test the *Co39/Pia* gene (Telebanco-Yanoria et al. 2011). Thus the increase of resistant lesion triggered by *Pi1* under high nitrogen pressure is likely an intrinsic property of this resistance gene and not of the Co39 genetic background. This also suggests that the differences of *R*-gene robustness under nitrogen are unlikely due to differences in the genetic background. This finding was confirmed by the observation that the *Pia* gene was efficient under high nitrogen in several genetic backgrounds tested (data not shown). While we never observe sporulation after breakdown of complete resistance triggered by the *R* genes tested, some *R* genes could be more prone to NIS than others. It is interesting to note that in the case of *Co39*/*Pia* (Figure 2C) and *Pi2* (data not shown), the effects of the *R* genes are visible before penetration while *Pi1* does not affect growth of the fungus before penetration. Whether these pre/post-penetration resistance phenotypes are related to the robustness of complete resistance under high nitrogen remains to be established.

### The NIS1 locus from Kasalath dominantly confers susceptibility under high nitrogen

Since the study by Talukder et al. (2005), there has been no report on the genetic analysis of NIS in rice. Here, using a CSSL population between Nipponbare and Kasalath, which show contrasted levels of NIS (Additional file 1: Figure S1 and S2), we identified a locus, called *NIS1*, on chromosome 1 (Figure 4A). We chose to further analyse this loci with F3 and F4 descendants because no other loci were identified with a strong confidence in the CSSL population. Analysis of heterozygous plants at this locus indicates that the Nipponbare parent carries a recessive allele of *NIS1* and conversely Kasalath a dominant allele conferring nitrogen-induced susceptibility (Figure 4B). As *R* genes are known to confer resistance in a dominant manner, *NIS1* is unlikely caused by an *R* gene from Nipponbare conferring robust resistance even under high nitrogen. Indeed, if this was the case, such an *R* gene from Nipponbare would likely confer robust resistance in heterozygous plants under high nitrogen. Rather the *NIS1* phenotype is more likely due to a gene from Kasalath that confers induced susceptibility under high nitrogen. The *NIS1* locus behaves like six of the loci previously identified (Talukder et al. 2005) as resistance is only lost under high nitrogen supply. When comparing the genetic maps, we found that *NIS1* could be allelic to the QTL 1.2 found by Talukder et al. (2005). This QTL 1.2 was defined by single-marker analysis and is linked to the C1370 marker which is physically inside the *NIS1* interval. However given the method used, the interval defining QTL 1.2 was not established and our work helps defining a small interval containing *NIS1* (Figure 4A).

Using available information on disease resistance in rice (Vergne et al. 2008), we found that region defining *NIS1* contains several categories of genes that could participate in disease resistance (Figure 4A): *NBS-LRR* and *WAK* genes that are involved in pathogen recognition, *MAPK* genes and *Rack1* involved in signal transduction and *WRKY* genes involved in transcriptional regulation of defense ((Delteil et al. 2010) and references herein). As discussed above, the heritability of *NIS1* suggests that *NBS-LRR* genes are unlikely involved. Similarly, it is unlikely that Kasalath alleles of positive regulators of disease resistance like WRKYs, Rack1 or MAPKs would confer dominant increase of susceptibility. On the opposite, these genes could be responsible for the *NIS1* phenotype if they encode for negative regulators of disease resistance and if high nitrogen increases their activity. To some extent this is possible since: (i) some *WRKY* transcription factors and *MAPKs* are negative regulators of disease resistance (Delteil et al. 2012; Xiong and Yang 2003) and (ii) nitrogen status can induce the expression of some *WKRY* genes (Gregersen and Holm 2007; Lin and Wu 2004). Mis-regulation by nitrogen metabolism of such negative regulators could affect the expression of defense-related genes and subsequent setting of quantitative resistance.

### A possible link between NIS and NUE

From the examination of genes involved in nitrogen metabolism, we found that the *NADH-GOGAT1* gene is located in the *NIS1* region (Figure 4A). The NADH-GOGAT enzyme is the glutamate synthase (EC 1.4.1.13) responsible of the production of L-glutamine and oxoglutarate by the recycling of glutamate and is thus a key element of the nitrogen assimilation pathway (Forde and Lea 2007; Tabuchi et al. 2007; Zhao and Shi 2006). The central role of the GS-GOGAT cycle in plant disease has been recently reviewed (Seifi et al. 2013). Nipponbare and Kasalath are known to be polymorphic for this enzymatic activity, with Kasalath displaying less NADH-GOGAT protein (Obara et al. 2000). Moreover, over-expression of a japonica allele of *GOGAT-NADH1* in the Kasalath background increased grain weight, indicating that this enzyme is limiting in this background for nitrogen utilization as compared to japonica cultivars (Yamaya et al. 2002). In that context, it is yet difficult to understand how the Kasalath allele of *NADH-GOGAT1* conferring less activity than the japonica one could dominantly affect susceptibility (Figure 4B). Unfortunately the transgenic lines already described (Yamaya et al. 2002) were lost during the 2011 earthquake in Japan. We tested the ARED12 mutant line available in Nipponbare from the OryzaTagLine library (Larmande et al. 2008) which has an insertion in the *NADH-GOGAT1* promoter but the expression of the gene was not affected by this insertion (data not shown). Thus the involvement of the *NADH-GOGAT1* gene in NIS remains to be clarified.

As suggested by the possible involvement of the *NADH-GOGAT1* gene in NIS, loci involved in the nitrogen physiology could be important. Rice displays a wide range of NUE levels (Koutroubas and Ntanos 2003) and this result was later reinforced by an independent study on 30 rice accessions (Namai et al. 2009). According to the later results, the Kasalath and Azucena genotypes have high NUE levels whereas other genotypes like Nipponbare, IR8 and IR64 have low NUE levels. Quite interestingly, these two sub-groups of rice lines show contrasted NIS levels (Figure 1A), with the genotypes with high NUE displaying high NIS levels and vice-versa. This suggests a possible link between NUE and NIS. However, the data from Trembese and Moroberkan do not support this relationship since they show low NIS and NUE levels comparable to Azucena. Nevertheless, these apparently conflicting data can be explained. First Trembese resistance is extremely high (Figure 1) and this does not allow proper analysis of nitrogen-induced susceptibility. Second, although it was not found to be significant, there was a 2.8-fold increase of susceptible lesions (Figure 1A) in Moroberekan. Considering these possible limitations, at this level of analysis the majority of the rice lines with high NUE levels are also the ones displaying high NIS. This is also consistent with the observation that the wheat Arche cultivar that shows high NUE compared to Récital (Le Gouis et al. 2000) is also showing the highest level of NIS (Figure 5). Quite interestingly, the *NIS1* locus is syntenic to a meta QTL controlling NUE in several Monocots (Quraishi et al. 2011). Thus NUE and NIS could be connected in the case of wheat and rice blast diseases.

## Conclusion

In this work we show that nitrogen-induced susceptibility affects both wheat and rice blast disease and that this phenomenon may be related to NUE. Moreover we show that complete resistance triggered by major resistance genes could also be affected. Finally, having identified rice recombinant lines and cultivars with altered NIS, it is now possible to evaluate how NIS under controlled conditions relates to well-known increased levels of disease in the field.

## Methods

### Plant growth, fertilization procedure, inoculation and disease assessment

Wheat and rice plants were sown on Wednesdays in Neuhaus S soil in which poudzolane was added (2 L/70L1N). Rice plants were grown as described previously (Faivre-Rampant et al. 2008) and wheat plants were grown at 24°C/20°C, 16 h light. Plants were grown under standard fertilization for three weeks and one day before inoculation, the 0 N and 1 N treatments were applied (See Additional file 1: Table S4 for detailed compositions). The 0 N solution contains the same fertilization elements than the 1 N solution except that it does not contain any source of nitrogen. The 1 N solution contains 50%NH4+/50%NO3- (40 mg/L). Four-week-old plants (4-leaf stage) were inoculated with spore suspensions as described in (Berruyer et al. 2003. For gene expression studies, we also used a mock treatment corresponding to the solution into which spores are re-suspended (*i.e.* 0.5% gelatin solution). Five to seven days after inoculation, the symptoms were scanned and the number of susceptible or resistant lesions (see arrows on Figure 1B) were counted using ImageJ software (http://rsbweb.nih.gov/ij/).

### DNA extraction and microsatellite analysis

CSSL and parent plants tissues were harvested and ground in liquid nitrogen. About 600 μl of powder was then treated with MATAB buffer (Tris HCl 1 M pH = 8, NaCl 4 M, EDTA 0.5 M pH = 8, Mixed AlkylTrimethyl Aminium Bromide 1%, NaSO4 0.25%) warmed at 65°C, incubated during 30 min at 65°C with shaking every 10 min. A volume of 600 μl of CHCl3 was then added and the samples were vortexed for 30 s. Supernatant was transferred into a new tube and 0.8 volume of isopropanol was added to precipitate DNA. The samples were then centrifuged (30 min, 3 000 rpm). DNA pellets were washed with 70% ethanol and resuspended in distilled water. The DNA for mapping were extracted using a KingFisher 96 machine (http://www.thermo.com) with LGC GENOMICS beadex Maxi Plant Kit (10 × 96) according to the manufacturer’s procedure (http://www.lgc.co.uk/).

Rice microsatellites markers were selected from Gramene database (http://www.gramene.org/; (International Rice Genome Sequencing Project 2005). Polymerase chain reactions (PCR) were performed in a volume of 20 μl in a PTC-100, MJ thermo cycler (MJ Research Inc, USA). The reaction mixture contained each primer at 0.5 μM, 200 μM deoxy nucleotide, Taq buffer, 2 U GoTaq Polymerase (Promega, Madison, WI, USA), 5 ng of DNA, completed with distilled water (Gibco) to 20 μL. After 5 min at 95°C, the PCR involved 35 cycles of amplification, each cycle comprising 30s at 95°C, 30s at 55°C, 30s at 72°C; and with a final extension step of 5 min at 72°C. Microsatellite amplicons were separated on 3% agarose gels (Sigma).

### Tissue staining for confocal observation

Inoculated plant tissues were harvested and fixed in fixation buffer (ethanol/CHCl3 3:1, 0.15% acetic acid) 18 h at room temperature. Fixed tissues were washed with ethanol 50% then put in distilled water. Tissues were cleared with NaOH 0.05M during 15 min at 90°C. Cleared tissues were washed two times with distilled water, then incubated with TrisHCl (0.1M, pH 5.8) during 30 min at 90°C, then washed again with distilled water and PBS 1X two times during 10 min. Fungal chitin in plant tissues was stained with WGA Alexa 488 (0.002%; Invitrogen) overnight in darkness at room temperature. Then, tissues were washed twice with distilled water and PBS 1X for 30 min. Polysaccharides of the plant cell wall were stained with Calcofluor white (0.001%; Sigma) overnight in darkness at room temperature. Then, tissues were washed twice with distilled water for 30 min. Stained tissues were stored in 50% glycerol. Observation were performed on LSM700 (ZEISS, http://www.zeiss.com) confocal microscope.

### RNA extraction and RT-PCR analysis

For RT-QPCR applications, frozen tissue was ground in liquid nitrogen. Approximately 500 μL of powder was then treated with 500 μL TLES buffer (Tris pH8 100 mM, LiCl 100 mM, EDTA pH8 10 mM and SDS 1%), 500 μL warmed water-saturated phenol (Gen-Apex®), and vortexed for 30 s. A volume of 500 μL of chloroform: isoamylalcohol (24:1) was then added and the samples were vortexed for 30 s. The samples were then centrifuged (30 min, 13 000 rpm) and the RNA was precipitated from the supernatant overnight using one volume LiCl 4 M. RNA pellets were then washed with 70% ethanol and resuspended in distilled water. RNA samples (5 μg) were denatured for 5 min at 65°C with oligo dT (3.5 μM) and dNTP (1.5 μM). They were then subjected to reverse transcription for 60 min at 37°C with 200 U of reverse transcriptase M-MLV (Promega, Madison, WI, USA), and DTT (5 mM) in 20 μL of the appropriate buffer. Two microliters of cDNA (dilution 1/30) were then used for quantitative RT-PCR. Quantitative RT-PCR mixtures contained forward and reverse primers (300 nM), and Roche qPCR SYBR Green Master Mix as per the manufacturer’s recommendations (Roche, Meylan, France). Amplification was performed as follows: 95°C for 10 min; 40 cycles of 95°C for 15 s, 62°C for 15 s and 72°C for 15 s; then 95°C for 1 min and 55°C for 30 s. The quantitative RT-PCR (QRTPCR) reactions were performed using a LightCycler^®^ 480 Real-Time PCR System (Roche) and data were extracted using the LC480 software. The amount of plant RNA in each sample was normalized using actin (Os03g50890) as internal control. PCR primers are provided in Additional file 1: Table S5.

### Statistical analysis

Distribution means were compared using the non parametric Kruskal-Wallis’ test in case of three and more distributions, and using the non-parametric Wilcoxon’s test in case of two distributions (at least one distribution was not normal in every comparison). All tests were performed using XLStat software (http://www.xlstat.com/, Addinsoft).

## Electronic supplementary material

Additional file 1: Figure S1.: Typical symptoms of Nitrogen-induced susceptibility. Nitrogen (1N) or no nitrogen (0N) was added to rice plants one day before inoculation with the indicated isolate of *M. oryzae*. Susceptible lesions were counted (Additional file 1: Figure S2). **Figure S2.** Nitrogen-induced susceptibility in CSSL4, 5 and 19 identifying the *NIS1* locus. Nitrogen (black bars; 1N) or no nitrogen (white bars; 0N) was added to rice plants one day before inoculation with the indicated isolate of *M. oryzae*. Susceptible lesions were counted 7 days post-inoculation. Statistical differences between 0N and 1N are shown (Wilcoxon tests; *: p<0.05; **p<0.01; ***: p<0.001). **Table S1.** NIS across rice and M.oryzae diversity. Symptoms of 14 rice varieties inoculated with 3 M. oryzae isolates. 4 weeks old plant were inoculated and symptoms were evaluated. Nitrogen or no nitrogen was added to rice plants one day before inoculation and the symptoms differences between both treatments were evaluated 7 days post-inoculation. **Table S2.** Recombinant lines establishing NIS1 on chromosome 1. Genotype of the recombinant lines at the SSR markers on chromosome 1 and on the other chromosome were residual Kasalath insertions were present based on published cartography of the parental population. **Table S3.** Further mapping of the NIS1 locus. Genotype of the recombinant lines on chromosome 1 establishing a fine mapping of NIS1 locus. **Table S4.** Composition of fertilizing solution. Nutritive elements used for the fertilizing solution added one day before inoculation: Nitrogen (1N) or no nitrogen (0N). **Table S5.** Primer sequences for q-PCR experiments. Sequences of the primer used to follow the expression of 8 defense genes. (PPTX 1 MB)

Below are the links to the authors’ original submitted files for images.

Authors’ original file for figure 1

Authors’ original file for figure 2

Authors’ original file for figure 3

Authors’ original file for figure 4

Authors’ original file for figure 5
