# Supplementary material for: Diversity and genetics of nitrogen-induced susceptibility to the blast fungus in rice and wheat
Source: Rice (N Y). 2013 Nov 20;6:32. doi: 10.1186/1939-8433-6-32 (PMC4883689; doi:10.1186/1939-8433-6-32)
Supplement: Supplementary file 1 — Additional file 1: Figure S1.: Typical symptoms of Nitrogen-induced susceptibility. Nitrogen (1N) or no nitrogen (0N) was added to rice plants one day before inoculation with the indicated isolate of M. oryzae. Susceptible lesions were counted (Additional file 1: Figure S2). Figure S2. Nitrogen-induced susceptibility in CSSL4, 5 and 19 identifying the NIS1 locus. Nitrogen (black bars; 1N) or no nitrogen (white bars; 0N) was added to rice plants one day before inoculation with the indicated isolate of M. oryzae. Susceptible lesions were counted 7 days post-inoculation. Statistical differences between 0N and 1N are shown (Wilcoxon tests; *: p<0.05; **p<0.01; ***: p<0.001). Table S1. NIS across rice and M.oryzae diversity. Symptoms of 14 rice varieties inoculated with 3 M. oryzae isolates. 4 weeks old plant were inoculated and symptoms were evaluated. Nitrogen or no nitrogen was added to rice plants one day before inoculation and the symptoms differences between both treatments were evaluated 7 days post-inoculation. Table S2. Recombinant lines establishing NIS1 on chromosome 1. Genotype of the recombinant lines at the SSR markers on chromosome 1 and on the other chromosome were residual Kasalath insertions were present based on published cartography of the parental population. Table S3. Further mapping of the NIS1 locus. Genotype of the recombinant lines on chromosome 1 establishing a fine mapping of NIS1 locus. Table S4. Composition of fertilizing solution. Nutritive elements used for the fertilizing solution added one day before inoculation: Nitrogen (1N) or no nitrogen (0N). Table S5. Primer sequences for q-PCR experiments. Sequences of the primer used to follow the expression of 8 defense genes. (PPTX 1 MB) [file 12284_2013_64_MOESM1_ESM.pptx]

## Slide 1
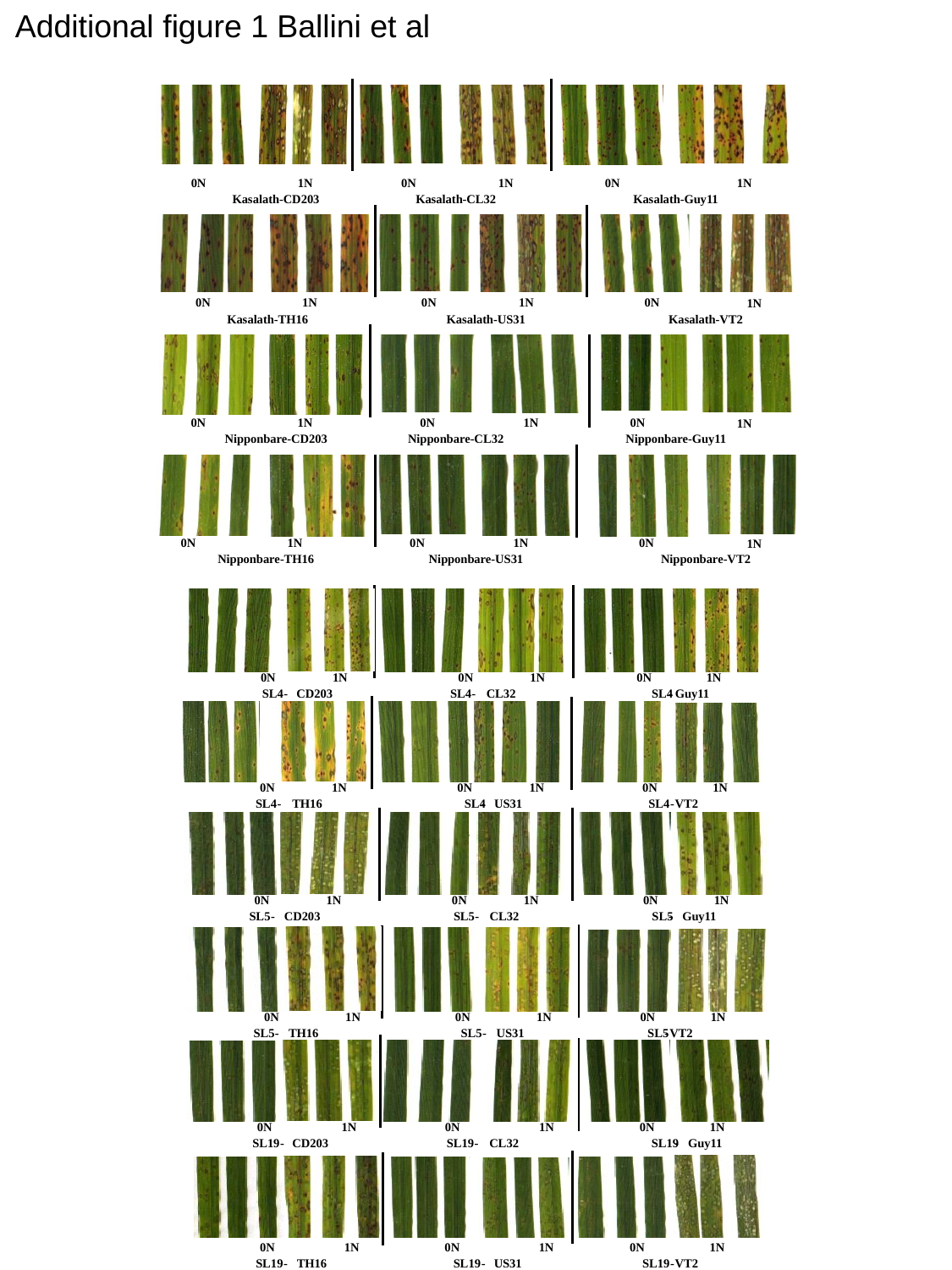

Additional figure 1 Ballini et al
0N 1N 0N 1N 0N
1N
Kasalath-CD203
Kasalath-CL32
Kasalath-Guy11
0N 1N 0N 1N 0N
1N
Kasalath-TH16
Kasalath-US31
Kasalath-VT2
0N 1N 0N 1N 0N
1N
Nipponbare-CD203
Nipponbare-CL32
Nipponbare-Guy11
0N 1N 0N 1N 0N
1N
Nipponbare-TH16
Nipponbare-US31
Nipponbare-VT2
0N 1N 0N 1N 0N
1N
SL4
-
CD203
SL4
-
CL32 SL4
-
Guy11
0N 1N 0N 1N 0N
1N
SL4
-
TH16 SL4
-
US31
SL4
-
VT2
0N 1N 0N 1N 0N
1N
SL5
-
CD203
SL5
-
CL32 SL5
-
Guy11
0N 1N 0N 1N 0N
1N
SL5
-
TH16
SL5
-
US31 SL5
-
VT2
0N 1N 0N 1N 0N
1N
SL19
-
CD203
SL19
-
CL32 SL19
-
Guy11
0N 1N 0N 1N 0N
1N
SL19
-
TH16
SL19
-
US31
SL19
-
VT2

## Slide 2
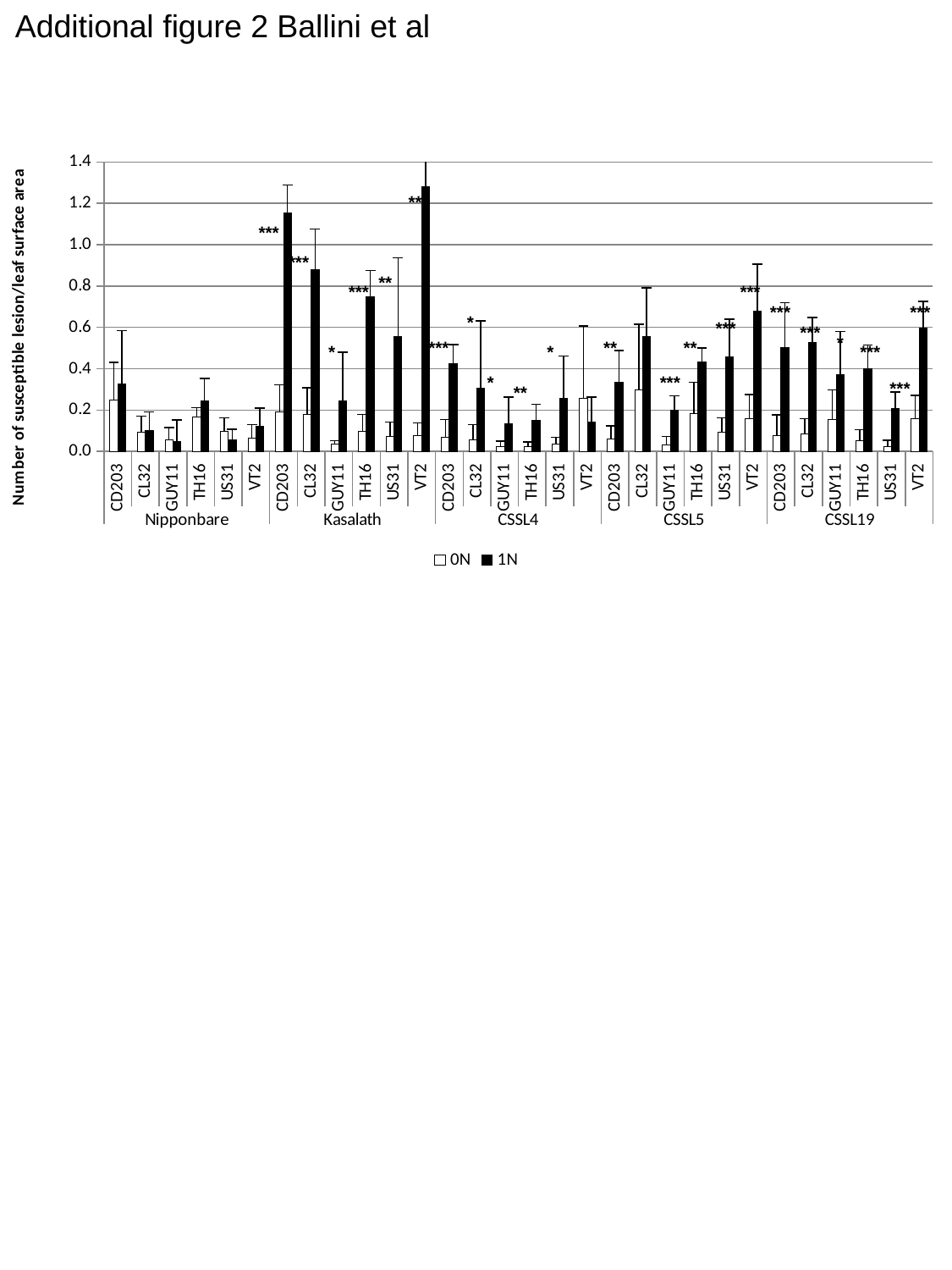

Additional figure 2 Ballini et al
### Chart
| Category | 0N | 1N |
|---|---|---|
| CD203 | 0.24653972862041998 | 0.3261359979564799 |
| CL32 | 0.09417401517020953 | 0.1017657200883461 |
| GUY11 | 0.057567461233709115 | 0.0490684688533502 |
| TH16 | 0.1643136178860699 | 0.24515126761848172 |
| US31 | 0.0966027465815005 | 0.05458671715309805 |
| VT2 | 0.06309366252190993 | 0.11934124884820328 |
| CD203 | 0.18994705066379883 | 1.15396692891138 |
| CL32 | 0.17888686953744792 | 0.8810164293452926 |
| GUY11 | 0.034041778233062675 | 0.2436616577427694 |
| TH16 | 0.0982892907915589 | 0.7491094879924557 |
| US31 | 0.07136480616667691 | 0.5578061074668292 |
| VT2 | 0.07630422776473848 | 1.2825499008302121 |
| CD203 | 0.06978605259709841 | 0.4264554422984848 |
| CL32 | 0.05378820089155392 | 0.3050706969888032 |
| GUY11 | 0.022725348311909366 | 0.1324483116069436 |
| TH16 | 0.02171986626084113 | 0.14831889000319584 |
| US31 | 0.03564847827819708 | 0.2586086180249472 |
| VT2 | 0.25456705315855355 | 0.1419031010438515 |
| CD203 | 0.05899012631715449 | 0.33304676863654653 |
| CL32 | 0.2980282229578126 | 0.5551596613369015 |
| GUY11 | 0.03245036227649349 | 0.19872763293541254 |
| TH16 | 0.18109929724238844 | 0.43267994937959103 |
| US31 | 0.09109364142311818 | 0.4565745648224754 |
| VT2 | 0.15767726664066584 | 0.6787605314813286 |
| CD203 | 0.0758364100111381 | 0.501917349013581 |
| CL32 | 0.08341313486993042 | 0.5276967501650337 |
| GUY11 | 0.15510163960528953 | 0.3720248781004191 |
| TH16 | 0.051526763194178996 | 0.39820271608127084 |
| US31 | 0.022188088411920052 | 0.20702086041445622 |
| VT2 | 0.15765539499779227 | 0.5979539229568486 |***
***
***
**
***
***
***
***
*
***
***
*
***
**
**
*
*
***
*
***
***
**

## Slide 3
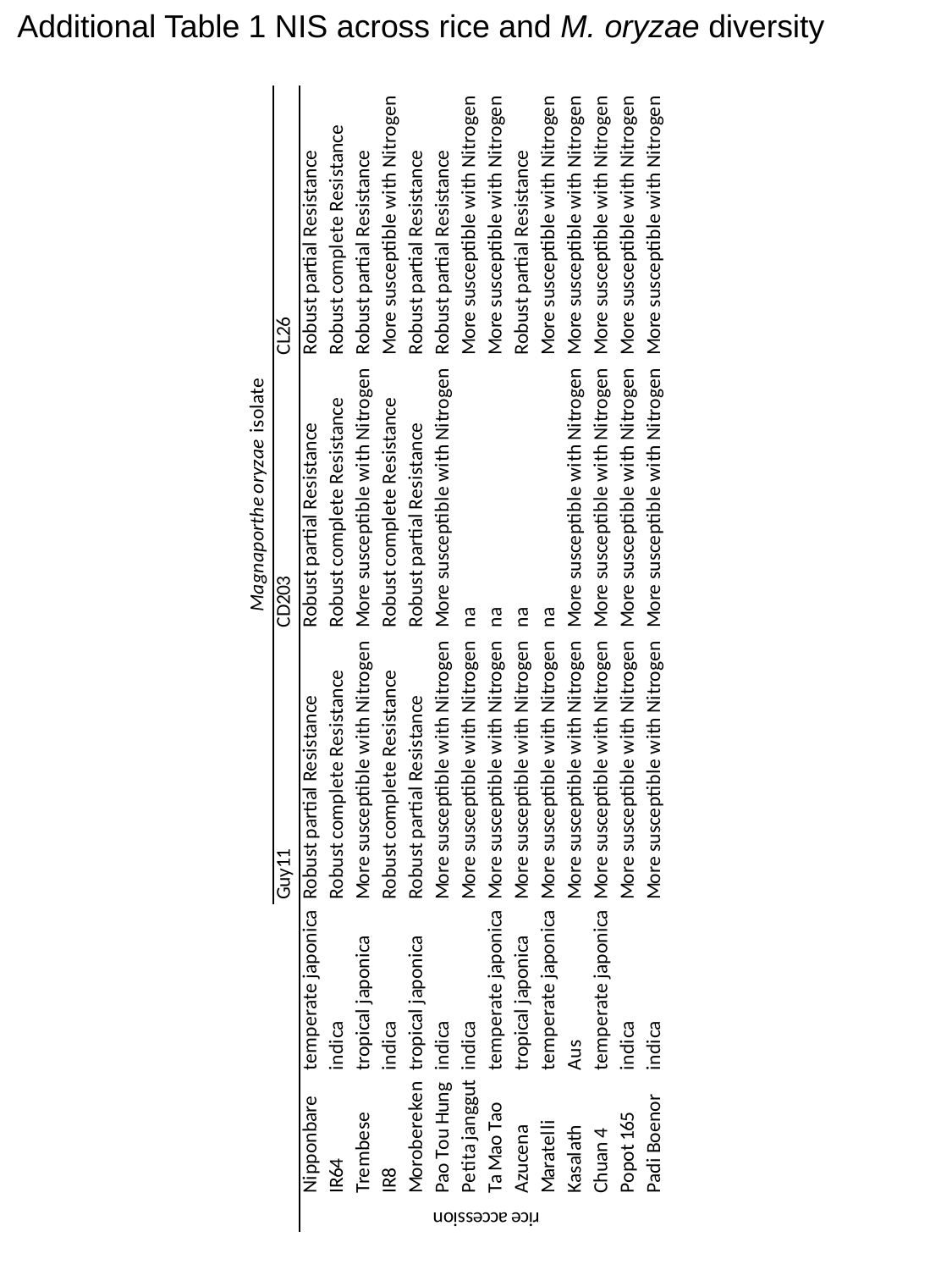

Additional Table 1 NIS across rice and M. oryzae diversity

## Slide 4
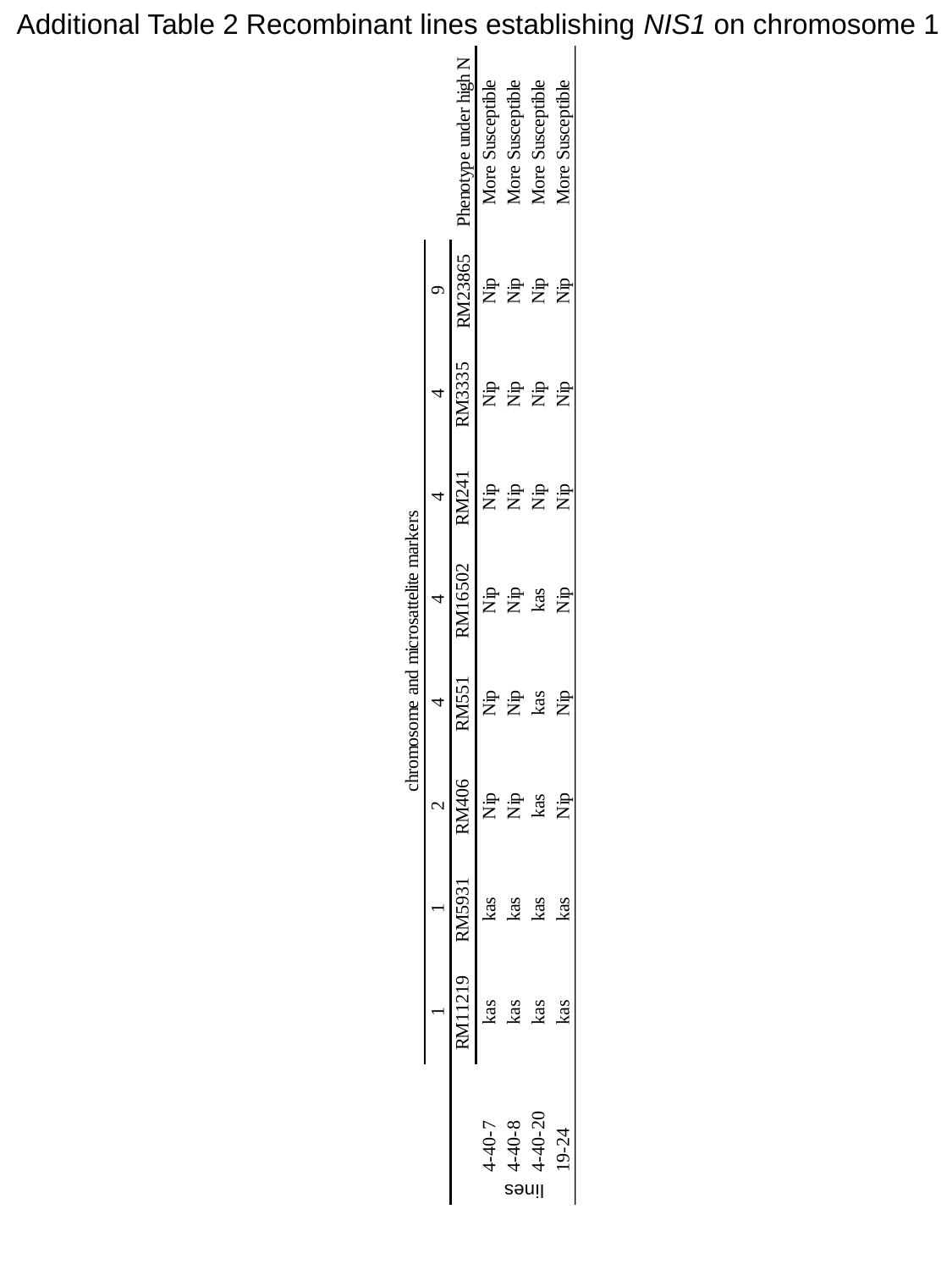

Additional Table 2 Recombinant lines establishing NIS1 on chromosome 1

## Slide 5
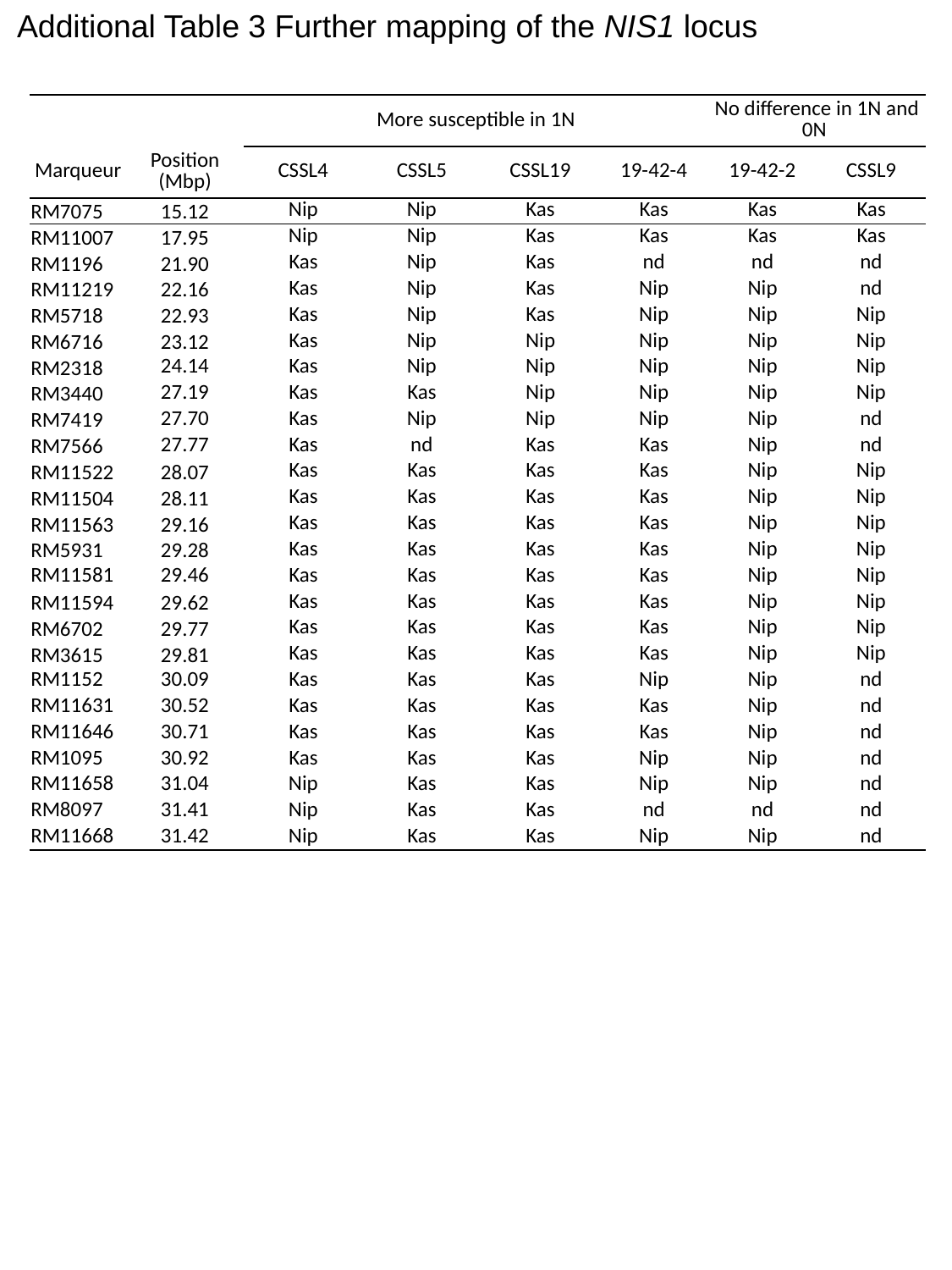

Additional Table 3 Further mapping of the NIS1 locus
| | | More susceptible in 1N | | | | No difference in 1N and 0N | |
| --- | --- | --- | --- | --- | --- | --- | --- |
| Marqueur | Position (Mbp) | CSSL4 | CSSL5 | CSSL19 | 19-42-4 | 19-42-2 | CSSL9 |
| RM7075 | 15.12 | Nip | Nip | Kas | Kas | Kas | Kas |
| RM11007 | 17.95 | Nip | Nip | Kas | Kas | Kas | Kas |
| RM1196 | 21.90 | Kas | Nip | Kas | nd | nd | nd |
| RM11219 | 22.16 | Kas | Nip | Kas | Nip | Nip | nd |
| RM5718 | 22.93 | Kas | Nip | Kas | Nip | Nip | Nip |
| RM6716 | 23.12 | Kas | Nip | Nip | Nip | Nip | Nip |
| RM2318 | 24.14 | Kas | Nip | Nip | Nip | Nip | Nip |
| RM3440 | 27.19 | Kas | Kas | Nip | Nip | Nip | Nip |
| RM7419 | 27.70 | Kas | Nip | Nip | Nip | Nip | nd |
| RM7566 | 27.77 | Kas | nd | Kas | Kas | Nip | nd |
| RM11522 | 28.07 | Kas | Kas | Kas | Kas | Nip | Nip |
| RM11504 | 28.11 | Kas | Kas | Kas | Kas | Nip | Nip |
| RM11563 | 29.16 | Kas | Kas | Kas | Kas | Nip | Nip |
| RM5931 | 29.28 | Kas | Kas | Kas | Kas | Nip | Nip |
| RM11581 | 29.46 | Kas | Kas | Kas | Kas | Nip | Nip |
| RM11594 | 29.62 | Kas | Kas | Kas | Kas | Nip | Nip |
| RM6702 | 29.77 | Kas | Kas | Kas | Kas | Nip | Nip |
| RM3615 | 29.81 | Kas | Kas | Kas | Kas | Nip | Nip |
| RM1152 | 30.09 | Kas | Kas | Kas | Nip | Nip | nd |
| RM11631 | 30.52 | Kas | Kas | Kas | Kas | Nip | nd |
| RM11646 | 30.71 | Kas | Kas | Kas | Kas | Nip | nd |
| RM1095 | 30.92 | Kas | Kas | Kas | Nip | Nip | nd |
| RM11658 | 31.04 | Nip | Kas | Kas | Nip | Nip | nd |
| RM8097 | 31.41 | Nip | Kas | Kas | nd | nd | nd |
| RM11668 | 31.42 | Nip | Kas | Kas | Nip | Nip | nd |

## Slide 6
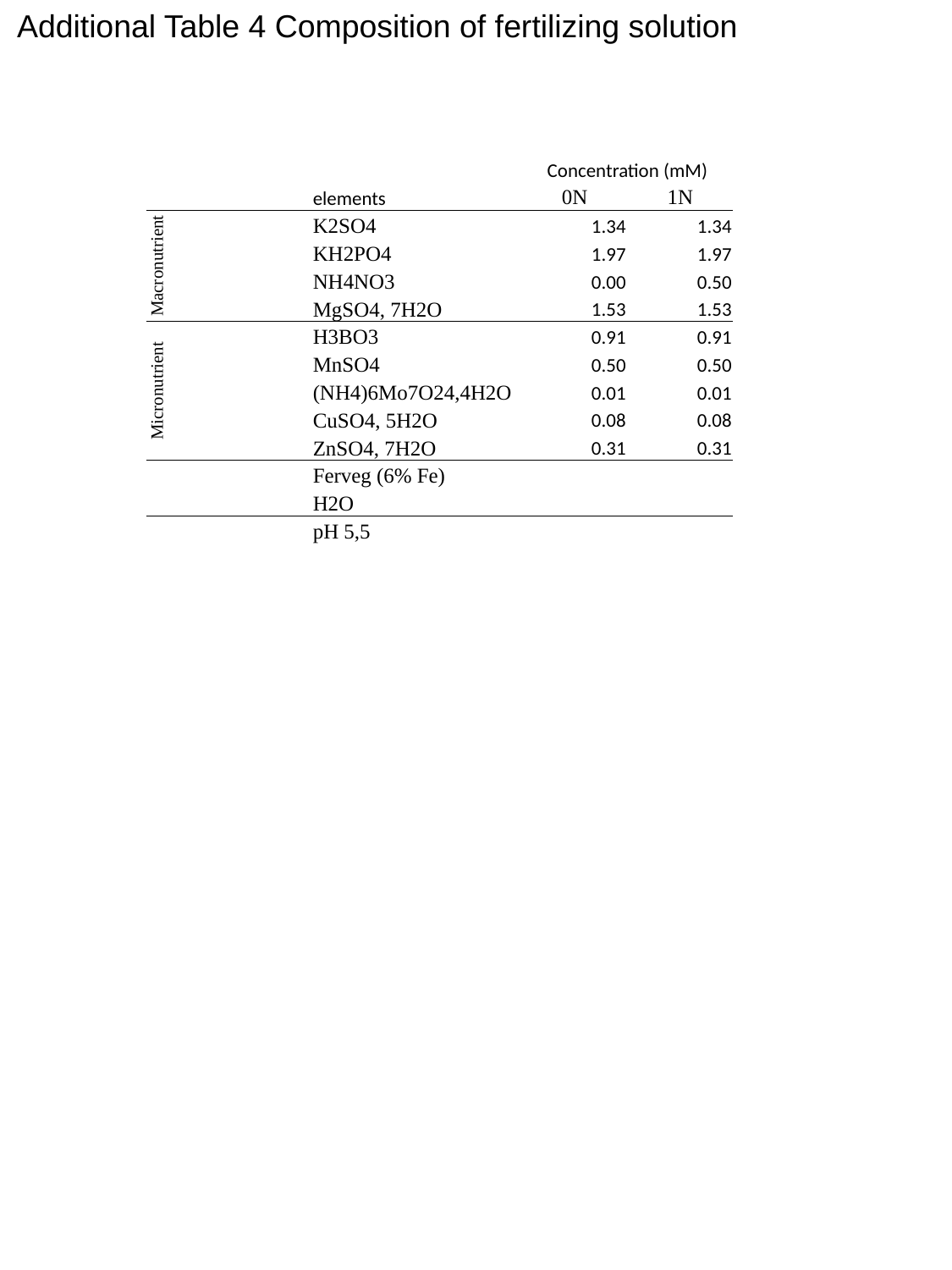

Additional Table 4 Composition of fertilizing solution
| | | Concentration (mM) | |
| --- | --- | --- | --- |
| | elements | 0N | 1N |
| Macronutrient | K2SO4 | 1.34 | 1.34 |
| | KH2PO4 | 1.97 | 1.97 |
| | NH4NO3 | 0.00 | 0.50 |
| | MgSO4, 7H2O | 1.53 | 1.53 |
| Micronutrient | H3BO3 | 0.91 | 0.91 |
| | MnSO4 | 0.50 | 0.50 |
| | (NH4)6Mo7O24,4H2O | 0.01 | 0.01 |
| | CuSO4, 5H2O | 0.08 | 0.08 |
| | ZnSO4, 7H2O | 0.31 | 0.31 |
| | Ferveg (6% Fe) | | |
| | H2O | | |
| | pH 5,5 | | |

## Slide 7
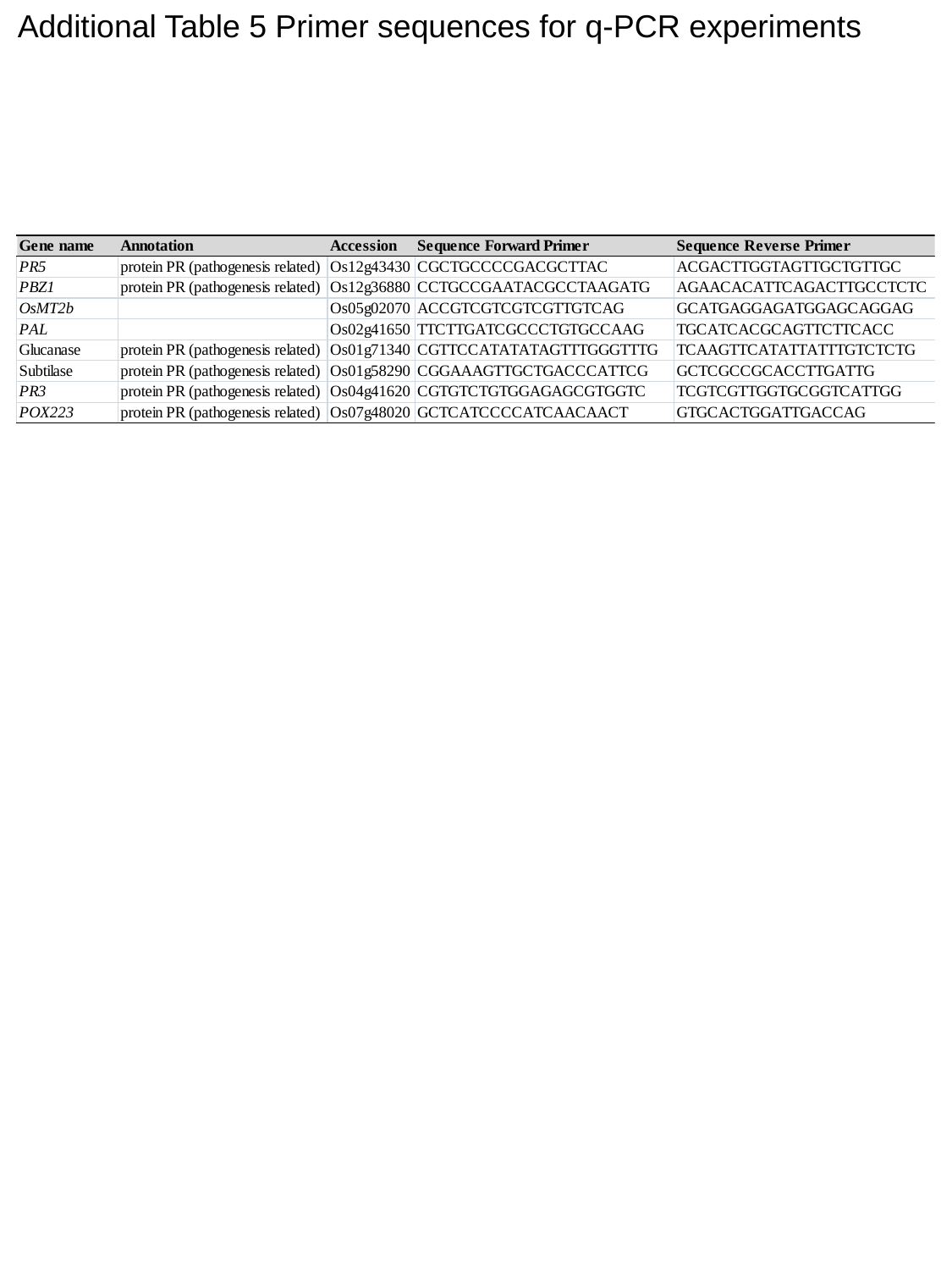

Additional Table 5 Primer sequences for q-PCR experiments
