# Supplementary figures and images for: Diversity and genetics of nitrogen-induced susceptibility to the blast fungus in rice and wheat
Source: Rice (N Y). 2013 Nov 20;6:32. doi: 10.1186/1939-8433-6-32 (PMC4883689; doi:10.1186/1939-8433-6-32)

**A**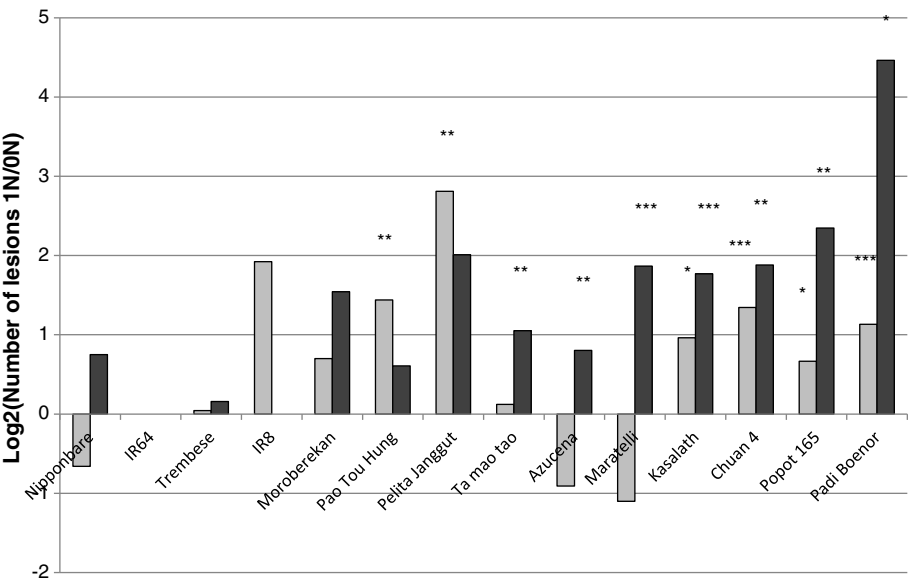**B**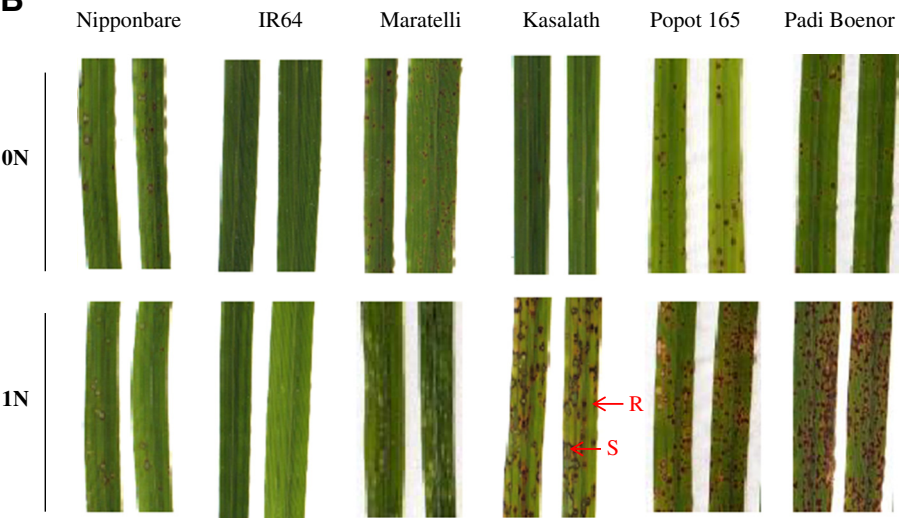

Supplement: Supplementary file 2 — Authors’ original file for figure 1 [file 12284_2013_64_MOESM2_ESM.pdf]

**Os12g36880 Pbz1**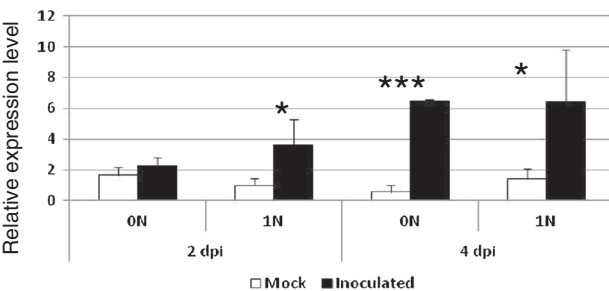**Os01g71340 Glucanase**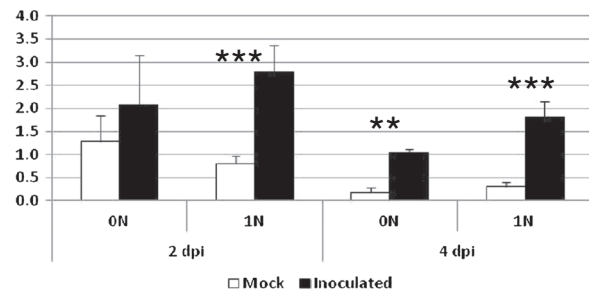**Os01g58290 Subtilase**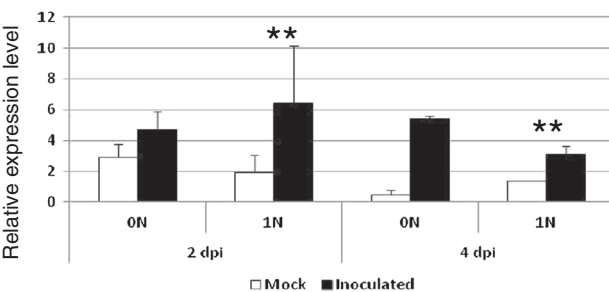**Os02g41680 PAL**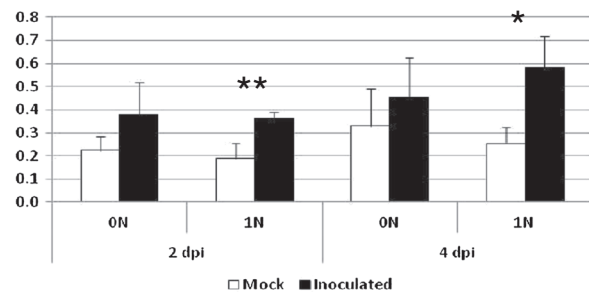**Os12g43430 PR5**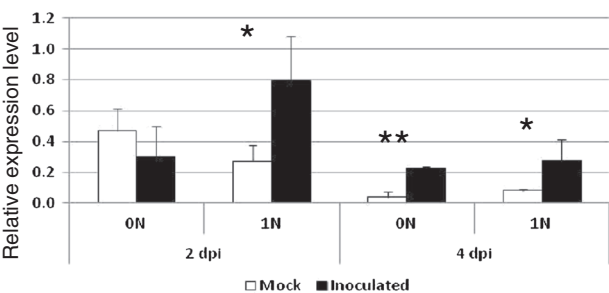**Os07g48020 POX223**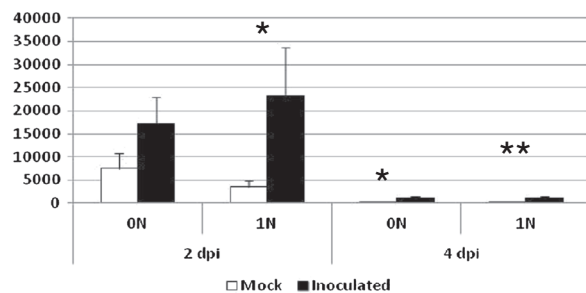**Os04g41620 PR3**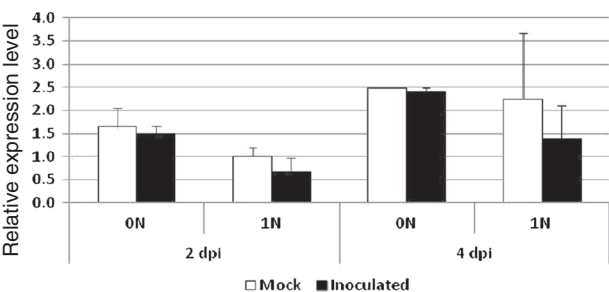**Os05g02070 OsMT2b**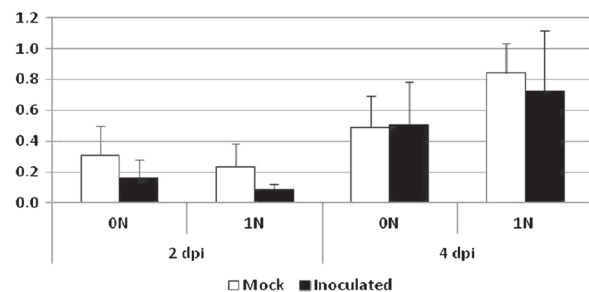

Supplement: Supplementary file 4 — Authors’ original file for figure 3 [file 12284_2013_64_MOESM4_ESM.pdf]

**A**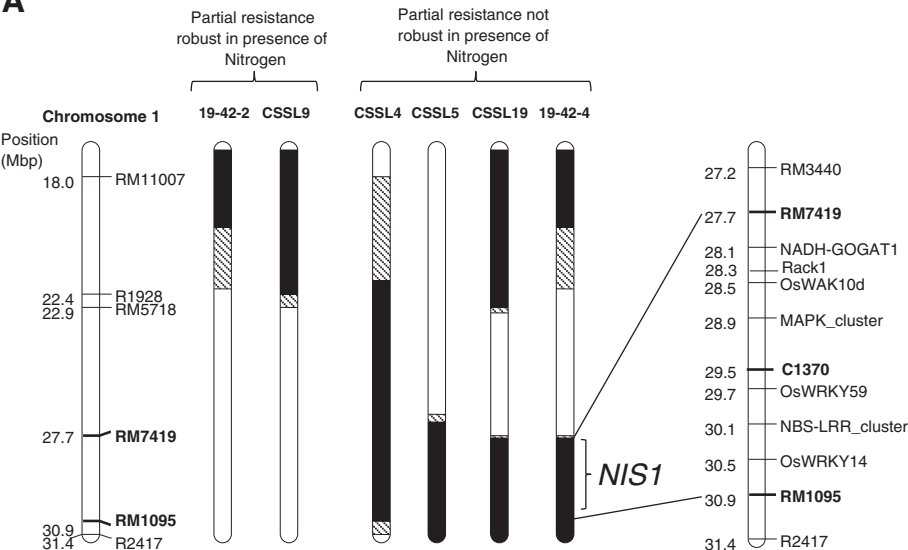**B**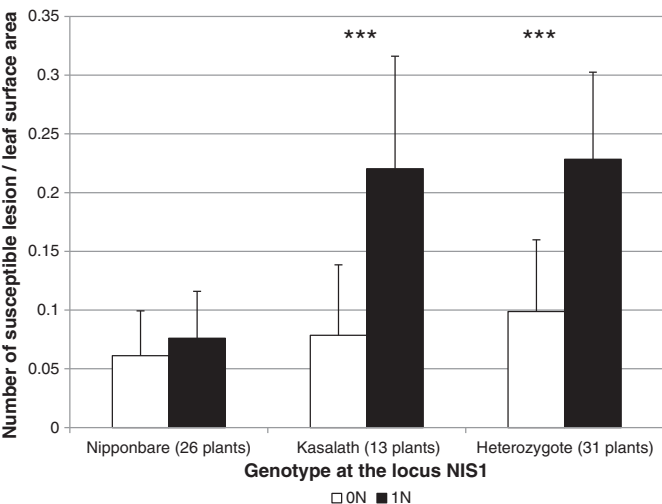

Supplement: Supplementary file 5 — Authors’ original file for figure 4 [file 12284_2013_64_MOESM5_ESM.pdf]

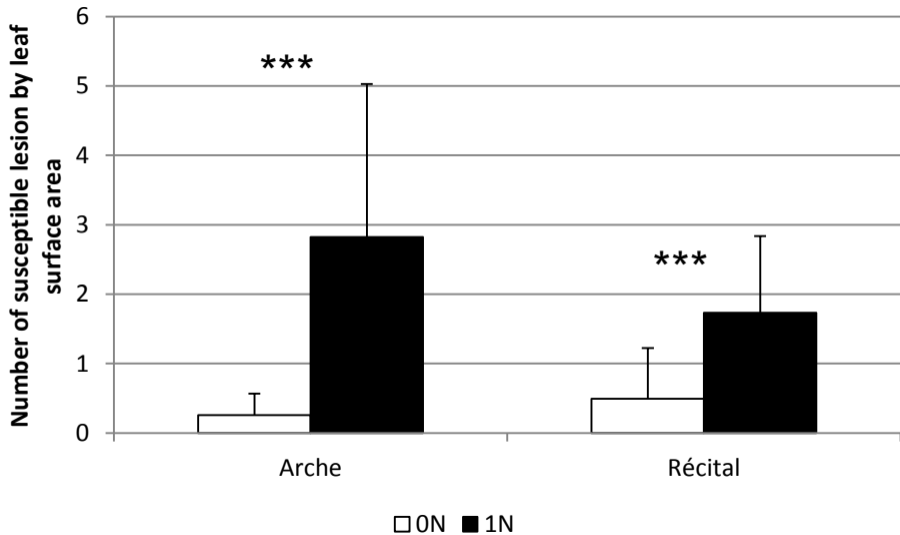

Supplement: Supplementary file 6 — Authors’ original file for figure 5 [file 12284_2013_64_MOESM6_ESM.pdf]
