# Supplementary material for: Diversity and genetics of nitrogen-induced susceptibility to the blast fungus in rice and wheat
Source: Rice (N Y). 2013 Nov 20;6:32. doi: 10.1186/1939-8433-6-32 (PMC4883689; doi:10.1186/1939-8433-6-32)
Supplement: Supplementary file 3 — Authors’ original file for figure 2 [file 12284_2013_64_MOESM3_ESM.pdf]

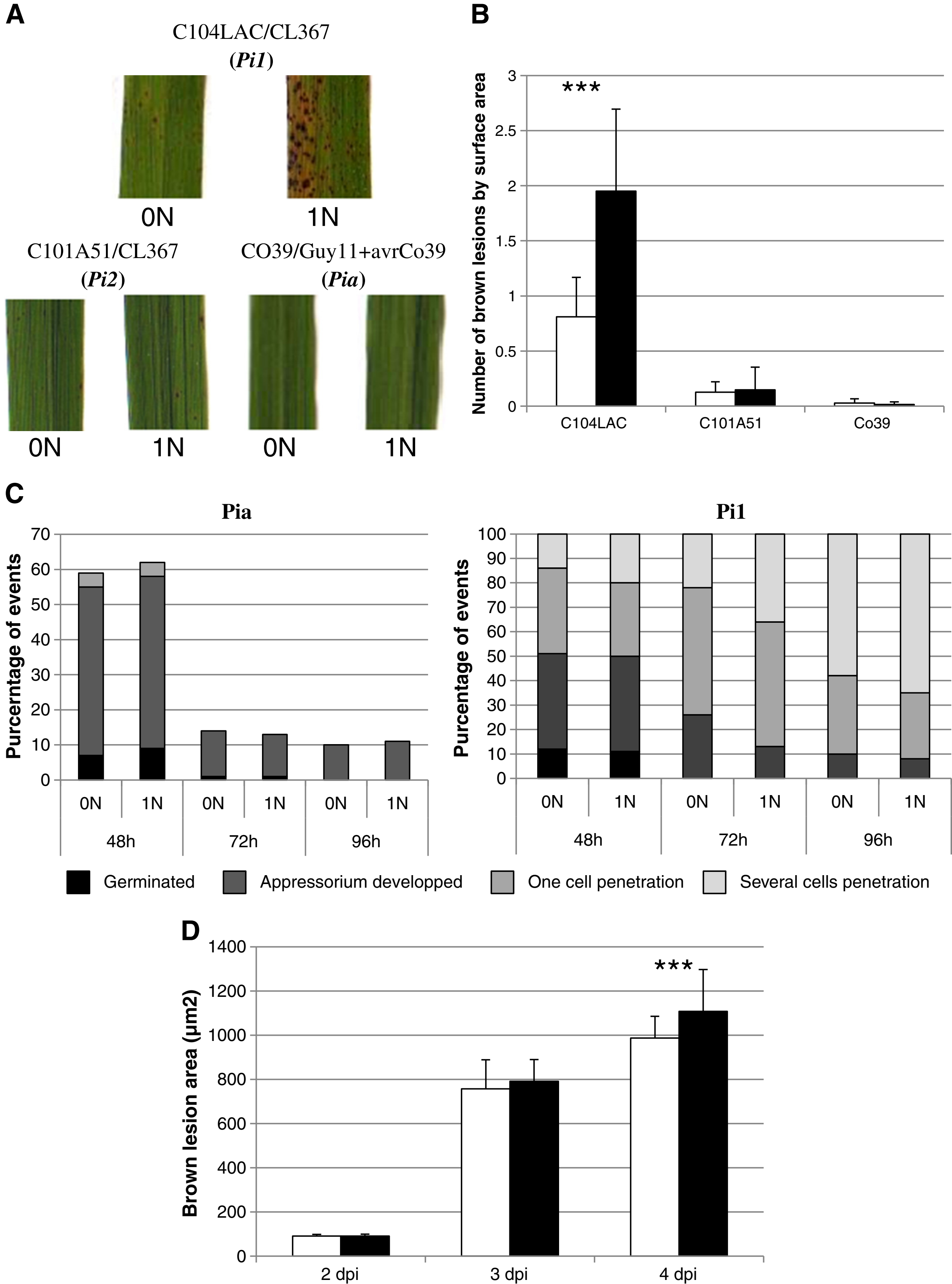

A
C104LAC/CL367
(Pi1)
0N
1N
C101A51/CL367
(Pi2)
CO39/Guy11+avrCo39
(Pia)
0N
1N
0N
1N
B
Number of brown lesions by surface area
***
C104LAC
C101A51
Co39
C
Pia
Pi1
Purcentage of events
0N
1N
48h
72h
96h
Germinated
Appressorium developped
One cell penetration
Several cells penetration
D
Brown lesion area (µm2)
***
2 dpi
3 dpi
4 dpi
